# The Hidden Drivers of New Employees’ Adaptive Performance in the Context of AI: The Role and Mechanisms of Workplace Fear of Missing Out

**DOI:** 10.3390/bs16050825

**Published:** 2026-05-20

**Authors:** Bingyao Li, Yongyue Zhu, Yuwei Zhang, Lifu Jin

**Affiliations:** 1 School of Management, Jiangsu University, Zhenjiang 212013, China; libingyao@outlook.com (B.L.); zyw1242773270@163.com (Y.Z.); jszjjlf@ujs.edu.cn (L.J.); 2 Office of Admissions and Career Services, Jiangsu Maritime Institute, Nanjing 211170, China

**Keywords:** workplace fear of missing out, cognitive reappraisal, role stress, adaptive performance, leader empathy, fuzzy-set qualitative comparative analysis

## Abstract

The rapid integration of artificial intelligence (AI) into workplace ecosystems is intensifying adaptation pressure for new employees. This study examines how Workplace Fear of Missing Out (WFMO) influences adaptive performance in this context. Methods: Drawing on Conservation of Resources Theory and the Emotion Regulation Process Model, a dual-path mediating model was tested using survey data from 442 new employees. Hierarchical regression, the Bootstrap method, and fuzzy-set qualitative comparative analysis (fsQCA) were employed. Results: WFMO is positively associated with adaptive performance. Role stress and cognitive reappraisal function as independent mediators in this relationship. Leader empathy positively moderates both direct relationships and indirect mediating pathways. Fuzzy-set qualitative comparative analysis reveals two distinct configurational paths to high adaptive performance. Conclusion: Workplace Fear of Missing Out can be transformed into adaptive behavior through resource mobilization and cognitive reappraisal mechanisms, with leader empathy serving as a critical contextual amplifier. These findings challenge the traditional view of workplace anxiety as uniformly detrimental and provide actionable insights for organizational management in technology-driven environments.

## 1. Introduction

With the ongoing advancement of the new technological revolution, digital transformation, and industrial integration, modern organizations are facing an increasingly complex and dynamic external environment (Song et al., 2021). The widespread adoption of large-scale artificial intelligence (AI) models is fundamentally reshaping contemporary workplace ecosystems (Qi & Xiao, 2020). AI applications, such as generative AI and intelligent decision-support systems, not only restructure workflows through real-time data monitoring and automated task allocation but also recalibrate employees’ adaptation criteria through algorithm-based performance evaluation mechanisms (Chi et al., 2024). For new employees, increased algorithmic transparency, coupled with intensified competition and information overload, is likely to induce cognitive overload, thereby exacerbating adaptation pressure during the early stage of role transition (B. He et al., 2022; S. Liu et al., 2015; Zhao et al., 2024). In this context, understanding the psychological adaptation process of new employees has become a critical issue that warrants focused scholarly attention.

In this context, understanding the psychological adaptation process of new employees has become a critical issue warranting focused scholarly attention. A dynamic work environment—characterized by frequent changes in job demands and resources driven by technological advancements and organizational shifts—fundamentally alters the nature of workplace adaptability requirements. In AI-driven contexts specifically, this dynamism manifests through algorithm-based task allocation, real-time data monitoring, and continuous skill updating, creating a work setting in which job demands and resources may fluctuate from day to day. Adaptive performance refers to an individual’s capacity to flexibly adjust cognition and behavior in response to changing environmental demands and is widely regarded as a key indicator of effective functioning in dynamic work settings. This capability is closely associated with individual career development outcomes and plays a crucial role in shaping organizational adaptability and competitiveness (H. B. Wei et al., 2018; B. He et al., 2024). Existing research on Adaptive Performance has primarily adopted individual- and environment-centered perspectives. At the individual level, studies have emphasized the roles of personality traits, such as openness and conscientiousness, and self-efficacy in predicting adaptive behaviors (X. T. Chen & Xiong, 2017; Tan et al., 2018). At the environmental level, scholars have highlighted contextual influences, including leadership styles and human resource management practices (X. Y. Liu & Zheng, 2024). However, much of this literature assumes a relatively stable technological context, which limits its applicability to AI-driven and rapidly evolving workplaces. Critically, prior research has predominantly examined adaptive performance through either individual-centered or environment-centered lenses in isolation, failing to capture how internal psychological states and external contextual resources jointly shape newcomer adaptation. This fragmented approach leaves unanswered the question of how new employees’ subjective anxiety experiences interact with leadership support to produce adaptive outcomes in dynamic settings.” When the pace of technological change surpasses individuals’ capacity for cognitive updating, new employees may experience heightened WFMO, which could function as a critical psychological interference factor in the development of Adaptive Performance.

WFMO is a prevalent psychological state characterized by anxiety arising from concerns about missing career development opportunities or critical work-related information (Shi et al., 2023). In the context of accelerated information flows and intensified competitive pressure driven by AI technologies, WFMO is particularly salient among new employees. Prior research has predominantly examined fear of missing out (FOMO) within social media contexts, documenting its detrimental effects on individuals’ physical and mental health, social behaviors, and tendencies toward social media addiction (Baker et al., 2016; Elhai et al., 2020a, 2020b; Malik et al., 2020; Y. Zhang et al., 2021). Emerging studies have extended this concept to organizational settings, demonstrating that WFMO can significantly predict compulsive message-checking behaviors, exacerbate work–family conflict and job burnout, and intensify stress experiences in digital workplaces (Budnick et al., 2020; Shi et al., 2024; Marsh et al., 2024). However, the rapid knowledge iteration enabled by AI technologies has fundamentally altered the nature of workplace adaptability demands. The widespread use of AI tools has accelerated knowledge acquisition and skill development processes, requiring new employees not only to cope with continuous learning pressures but also to remain vigilant to environmental changes to avoid marginalization. Under such conditions, WFMO and the coping strategies it evokes may, paradoxically, facilitate employee adaptation. Nevertheless, existing research has yet to establish a systematic theoretical framework to explain this potential adaptive function. More specifically, three critical gaps remain. First, prior FOMO research has predominantly focused on its detrimental effects in social media contexts (Baker et al., 2016; Elhai et al., 2020a, 2020b; Malik et al., 2020), neglecting its potential motivational function in organizational settings. Second, existing studies have examined anxiety-to-performance relationships through single theoretical lenses—either stress-based (LePine et al., 2004; N. P. Podsakoff et al., 2007) or emotion-regulation-based perspectives (Gross, 1998; Goldin et al., 2009)—without integrating these perspectives. Third, the boundary conditions under which WFMO transforms from a hindrance to a resource remain insufficiently explored, particularly the role of leadership in this transformation process (Budnick et al., 2020; Marsh et al., 2024).

To address these gaps, this study proposes an integrated dual-path model that advances prior research in three ways. First, rather than treating workplace anxiety as uniformly detrimental, it examines when fear of missing out catalyzes adaptive performance. Second, instead of relying on single theoretical lenses, it integrates the Conservation of Resources Theory with the Emotion Regulation Process Model to simultaneously capture resource mobilization (role stress) and resource management (cognitive reappraisal). Third, beyond viewing leadership as a direct predictor, it positions leader empathy as a contextual amplifier of anxiety-to-adaptation transformation. This design is superior because it captures the dynamic interplay between internal psychological processes and external social resources, explaining how anxiety can be harnessed rather than merely managed. Conservation of Resources Theory emphasizes the dynamic balance of individual resources under stress (Halbesleben et al., 2014). From this perspective, the environmental uncertainty induced by AI technologies is not solely a psychological burden. When new employees perceive pressures related to skill iteration and explicit competitive threats, moderate WFMO may activate resource vigilance through role stress, motivating proactive engagement in AI tool learning and cross-domain skill development. This process forms a facilitative chain of “anxiety–resource mobilization–adaptive performance enhancement,” challenging the traditional assumption that role stress is uniformly detrimental and highlighting its goal-directed value during technological adaptation. Concurrently, the Emotion Regulation Process Model provides a theoretical basis for understanding how the emotional valence of anxiety can be strategically transformed. Faced with information overload and algorithmic transparency, new employees may engage in cognitive reappraisal—reframing “fear of missing out” as an “opportunity radar”—thereby optimizing the efficiency of resource allocation (Gross, 1998). This pathway becomes particularly critical in contexts where the pace of technological change exceeds individuals’ baseline adaptability, rendering cognitive transformation a cornerstone of effective resource management. These two theoretical perspectives are complementary rather than competing, jointly constituting a dual mechanism through which WFMO affects adaptive performance via resource mobilization (role stress pathway) and resource management (cognitive reappraisal pathway). Furthermore, leaders’ empathy, as a core organizational variable, may exert a moderating influence through a dual function: alleviating negative interpretations of role stress via emotional support and enhancing the effectiveness of cognitive reappraisal through role modeling and strategic guidance (X. Y. Liu & Wan, 2016). In AI-dominated workplaces characterized by heightened uncertainty, leaders’ empathetic behaviors can buffer the psychological rigidity imposed by algorithmic systems while offering practical direction for resource mobilization and strategic adjustment, thereby fostering a dynamic balance between technological demands and human needs. Accordingly, this study integrates AI context with resource preservation and emotional regulation perspectives to examine how WFMO influences new employees’ adaptive performance, aiming to advance theoretical understanding and provide actionable insights for supporting newcomer adaptation in rapidly evolving work environments. Specifically, this study seeks to address the following questions: (1) How is WFMO associated with the adaptive performance of new employees? (2) Do role stress and cognitive reappraisal function as transmission mechanisms between WFMO and adaptive performance? (3) Does leaders’ empathy moderate these relationships by strengthening the effectiveness of role stress and cognitive reappraisal? These drivers are considered “hidden” because workplace fear of missing out, as a psychological state, often operates beneath the surface of observable behaviors, subtly shaping new employees’ adaptive responses without being explicitly recognized by either the employees themselves or their organizations.

## 2. Theoretical Basis and Research Hypothesis

### 2.1. Workplace Fear of Missing Out and New Employee Adaptive Performance

Workplace Fear of Missing Out (WFMO) refers to a prevalent form of anxiety experienced by employees who worry about missing work-related information, social connections, career development opportunities, or job-related benefits (Shi et al., 2023). This anxiety typically arises from heightened sensitivity to organizational dynamics, uncertainty regarding career prospects, and a strong desire to maintain interactive relationships with colleagues. Prior research has indicated that prolonged WFMO may exert negative effects on employees’ physical and mental health as well as work performance, including diminished work attention and reduced well-being (Fridchay & Reizer, 2022). However, more recent studies have begun to explore the multidimensional effects of WFMO across different contexts. In rapidly changing work environments, its potential motivational functions have gradually attracted scholarly attention (Gartner et al., 2022). Although WFMO is generally conceptualized as a negative emotion, evidence suggests that, within a moderate range, it may stimulate greater initiative and work engagement. This effect appears particularly salient among new employees, for whom moderate anxiety may prompt proactive behaviors aimed at reducing information gaps and uncertainty. Budnick et al. noted that WFMO can activate employees’ motivation to acquire resources, thereby increasing their focus on work performance and career development (Budnick et al., 2020). Specifically, this emotional state may motivate new employees to engage more actively in team communication, knowledge acquisition, and skill development to better adapt to dynamic workplace demands. Through such processes, new employees may not only integrate into the organization more efficiently but also enhance their adaptive performance.

The concept of adaptive performance was first proposed by Allworth and Hesketh. Unlike traditional static performance indicators, adaptive performance emphasizes employees’ effectiveness in responding to continuously changing work environments (Griffin et al., 2007). Specifically, adaptive performance refers to employees’ capacity to flexibly adjust their behaviors, strategies, and attitudes to support workplace changes, reflecting a dynamic developmental process (Pan & Sun, 2018). In the context of AI-driven workplaces, adaptive performance has become increasingly critical, as AI technologies accelerate the frequency and unpredictability of job changes (Manning & Aguirre, 2026). Moreover, adaptive performance plays a particularly important role in the early socialization and adjustment of new employees. It not only enables individuals to cope with uncertainty and stress but also facilitates the establishment of new social networks and adaptation to organizational culture (Pulakos et al., 2000). Although existing research has extensively examined the antecedents of adaptive performance—focusing mainly on leadership styles, job characteristics, and motivational factors—relatively little attention has been paid to the role of WFMO from the perspective of individual psychological perception. For new employees, unfamiliar job tasks and novel organizational environments often evoke heightened insecurity and uncertainty during the initial career stage. In this context, adaptive performance serves a critical function by helping newcomers adjust more rapidly, build social relationships, enhance their understanding of organizational climates, and reduce perceived uncertainty.

According to Conservation of Resources Theory, individuals invest psychological and behavioral resources to meet adaptation demands when confronting new environments (Halbesleben et al., 2014). For new employees, WFMO reflects concerns about potential resource loss while simultaneously activating motivation for resource mobilization and accumulation. This motivation encourages proactive adaptation behaviors, such as information seeking, relationship building, and skill enhancement, which facilitate adjustment to the new organizational context. From a resource gain perspective, WFMO—when maintained at a moderate level—may activate resource acquisition behaviors that help new employees better satisfy adaptation needs, thereby enhancing their adaptive performance. Although extensive research has documented negative effects of FOMO (e.g., burnout, stress), moderate WFMO in AI-driven contexts may paradoxically motivate adaptive behaviors. The proposed positive association between WFMO and adaptive performance, while seemingly paradoxical, is theoretically grounded in the distinction between hindrance and challenge stressors. Although WFMO entails anxiety about potential losses, it does not necessarily overwhelm new employees’ coping resources. Instead, moderate WFMO can function as a challenge stressor that signals meaningful environmental changes—such as skill obsolescence risks or information gaps—thereby motivating proactive resource investment (LePine et al., 2004). This logic is particularly salient in AI-driven workplaces, where the pace of technological iteration substantially raises the cost of inattention. Under such conditions, the motivational benefits of moderate WFMO are likely to outweigh its psychological costs, transforming anxiety into a driver of adaptive behavior. Therefore, rather than assuming a uniformly detrimental effect, we propose a conditional positive association between WFMO and adaptive performance. Based on this reasoning, the present study proposes the following hypothesis:

**H1.**  *WFMO is positively associated with new employees’ adaptive performance.*

### 2.2. The Mediating Role of Role Stress

Role stress refers to the psychological strain experienced by individuals when they lack clarity about role-related tasks, perceive an inability to meet role expectations, or struggle to balance multiple role demands within an organization. It typically comprises three dimensions: role ambiguity, role conflict, and role overload (Peterson et al., 1995). Role ambiguity reflects a mismatch between the information received by role incumbents and the information required to perform their roles effectively; role conflict refers to incompatibility among competing role expectations; and role overload arises when role demands exceed an individual’s capacity. Fundamentally, role stress represents an imbalance between role expectations and available individual resources. When organizational task demands surpass new employees’ cognitive, emotional, or skill-based reserves, resource depletion is likely to occur, thereby triggering stress perceptions. Existing research has largely emphasized the detrimental effects of role stress. However, Conservation of Resources Theory suggests that individuals may engage in active resource investment strategies when confronted with resource threats, such as replenishing cognitive resources through skill acquisition or expanding social resources via network building. LePine et al. found that challenging stressors, including role ambiguity and role conflict, can stimulate employees’ intrinsic motivation and learning behaviors, ultimately enhancing job performance and adaptive performance (Hobfoll, 1989; LePine et al., 2004). Similarly, a meta-analysis by Podsakoff et al. demonstrated that, under moderate conditions, challenging stressors may exert positive effects on various dimensions of employee performance (N. P. Podsakoff et al., 2007). Accordingly, this study posits that WFMO may heighten new employees’ perceptions of role stress.

The core premise of Conservation of Resources Theory is that individuals are motivated to acquire, protect, and cultivate valuable resources (Halbesleben et al., 2014). When individuals perceive actual or potential resource loss, stress emerges and subsequently activates motivation to safeguard existing resources and obtain new ones. Importantly, resource acquisition and protection are viewed as dynamic processes (Hobfoll, 1989). In organizational settings, new employees who perceive the risk of missing opportunities may invest substantial time and effort to avoid marginalization, which can accelerate the consumption of limited resources such as energy and attention, thereby intensifying role stress. In response to heightened role stress, new employees are likely to adopt proactive behaviors aimed at acquiring and preserving critical resources. These behaviors include actively developing interpersonal networks to gain information and social support, as well as enhancing professional skills to increase personal value within the organization. Such resource-oriented actions facilitate more effective adaptation to the workplace environment. From this perspective, moderate role stress should not be regarded solely as a detrimental condition but rather as a form of challenging pressure that promotes resource accumulation and personal growth. In the short term, this pressure may enhance adaptability, while in the long term it may contribute to sustained career development. Based on the above reasoning, this study proposes the following hypothesis:

**H2.**  *Role stress is indirectly associated with the positive relationship between WFMO and new employees’ adaptive performance.*

### 2.3. The Mediating Role of Cognitive Reappraisal

Cognitive reappraisal is an adaptive emotion regulation strategy through which individuals regulate emotional responses by modifying their interpretations and evaluations of events (Gross, 1998). By reinterpreting the meaning and significance of situations, cognitive reappraisal enables individuals to cope more effectively with negative emotions and stress, thereby enhancing subjective well-being and functional adjustment (M. Chen et al., 2023). Existing research indicates that cognitive reappraisal is closely associated with a wide range of mental health outcomes and behavioral patterns (Gross & John, 2003). Individuals with higher levels of cognitive reappraisal tend to manage stressors more effectively, experience lower anxiety immersion, and maintain stronger task focus. In contrast, individuals with higher trait anxiety are less likely to employ cognitive reappraisal strategies and are more prone to entering cycles of emotional exhaustion and behavioral avoidance (M. Chen et al., 2023). Notably, WFMO, as a form of situational anxiety, shares certain persistence-related characteristics with trait anxiety. New employees often experience sustained tension due to ongoing concerns about missing critical information or career opportunities, which may constrain their spontaneous use of cognitive reappraisal strategies. However, the emotion regulation process model suggests that even under stressful conditions, individuals can deliberately select cognitive reappraisal to reconstruct their understanding of anxiety-provoking stimuli, thereby transforming emotional arousal into adaptive behavioral motivation (J. Gross, 1998).

According to the process model of emotion regulation, emotion regulation strategies can be deployed at different stages of emotional generation. Cognitive reappraisal, as an antecedent-focused strategy, mitigates negative emotional experiences by altering individuals’ cognitive representations of emotional events, enabling more constructive interpretations of situations that typically elicit frustration, loss, or threat (Wang et al., 2007). In the context of WFMO, excessive concerns about potential losses may intensify negative affect, such as anxiety and unease among new employees. Through cognitive reappraisal, perceived threats of resource loss can be reframed as opportunities for self-improvement and growth by redefining the meaning of these events (Goldin et al., 2009). For instance, failing to participate promptly in team discussions may be reinterpreted as a signal to enhance proactive communication, while pressure stemming from technological change may be reframed as a catalyst for accelerated professional development. Such cognitive restructuring not only attenuates the intensity of negative emotions but also releases psychological resources previously occupied by anxiety, allowing individuals to redirect their efforts toward adaptive behaviors such as skill acquisition and social interaction (Bell & Hall, 1954). Moreover, cognitive reappraisal enhances emotional flexibility, enabling new employees to maintain adaptive adjustment capacities in dynamic work environments. When WFMO induces emotional fluctuations, cognitive reappraisal facilitates rapid recalibration of stress appraisals, preventing rigid loss-oriented mindsets and fostering exploratory and learning-oriented behaviors. Based on the above reasoning, this study proposes the following hypothesis:

**H3.**  *Cognitive reappraisal is indirectly associated with the positive relationship between WFMO and new employees’ adaptive performance.*

### 2.4. The Moderating Effect of Leaders’ Empathy Ability

Leader empathy refers to leaders’ capacity to accurately perceive and understand employees’ emotional states, identify their underlying needs, and respond in a timely and appropriate manner (X. Y. Liu & Wan, 2016). It plays a critical role in facilitating new employees’ adaptation to organizational environments and in helping them cope with workplace challenges. From the Conservation of Resources Theory, WFMO may lead new employees to excessively attend to potential resource loss, thereby intensifying psychological and behavioral resource investment. Within this process, leader empathy can influence employees’ role stress and cognitive reappraisal through distinct mechanisms. Although empathy is generally regarded as a stress-buffering resource, leaders with high levels of empathy may, in certain situations, inadvertently elevate employees’ perceived performance expectations, thereby increasing role stress (Schaumberg & Flynn, 2017). When employees perceive strong empathetic concern from their leaders, they may experience heightened attention and expectations, which can motivate them to reciprocate through increased effort while simultaneously generating concern about failing to meet these expectations. Schaumberg and Flynn found that leaders’ empathetic attention may enhance employees’ performance motivation, but it can also heighten sensitivity to role demands, indirectly intensifying role stress (Schaumberg & Flynn, 2017). Importantly, this form of role stress is not purely detrimental; rather, within a moderate range, it may function as a challenging stressor that enhances employees’ sense of responsibility and motivates proactive behavioral adjustment. In contrast, leaders with high empathy are also more capable of recognizing employees’ emotional difficulties and facilitating more effective cognitive reappraisal through emotional support and guidance (W. Liu et al., 2010). By providing reassurance, feedback, and perspective-taking cues, empathetic leaders can help employees reinterpret WFMO as a developmental signal rather than a direct threat. For example, by fostering a supportive climate and offering constructive feedback, empathetic leaders may encourage employees to adopt cognitive reappraisal strategies and approach adaptation challenges more positively. Accordingly, leaders’ empathy may serve as a key contextual factor enabling employees to flexibly deploy cognitive reappraisal when confronting workplace stressors. Based on this reasoning, the following hypotheses are proposed:

**H4a.**  *Leaders’ empathy positively moderates the relationship between new employees’ WFMO and role stress, such that the relationship is stronger when leaders’ empathy is high.*

**H4b.**  *Leaders’ empathy positively moderates the relationship between new employees’ WFMO and cognitive reappraisal, such that the relationship is stronger when leaders’ empathy is high.*

In summary, leaders’ empathetic ability plays a crucial moderating role in the relationships among WFMO, role stress, cognitive reappraisal, and adaptive performance among new employees. Leaders with high empathy may function as important psychological and instrumental resources, supporting newcomers’ adjustment and enabling more effective responses to workplace challenges. While empathetic support can alleviate anxiety and enhance cognitive reappraisal capacity, it may simultaneously increase role stress by elevating perceived expectations and self-imposed performance standards. Consequently, variations in perceived leader empathy may lead to different adaptive pathways. Specifically, when new employees perceive high leader empathy, WFMO is more likely to activate cognitive reappraisal, thereby enhancing adaptive performance. At the same time, under conditions of high WFMO and high perceived leader empathy, new employees may also experience elevated role stress due to heightened expectations, motivating them to protect remaining resources by sustaining stable and high-level performance to maintain leaders’ trust and support. This motivation may drive increased effort, improved work quality, and enhanced efficiency, ultimately contributing to higher adaptive performance. Based on these arguments, this study proposes the following hypotheses:

**H5a.**  *Leaders’ empathy positively moderates the mediating effect of role stress on the relationship between WFMO and adaptive performance, such that the mediating effect is stronger when leaders’ empathy is high.*

**H5b.**  *Leaders’ empathy positively moderates the mediating effect of cognitive reappraisal on the relationship between WFMO and adaptive performance, such that the mediating effect is stronger when leaders’ empathy is high.*

**Figure 1:**
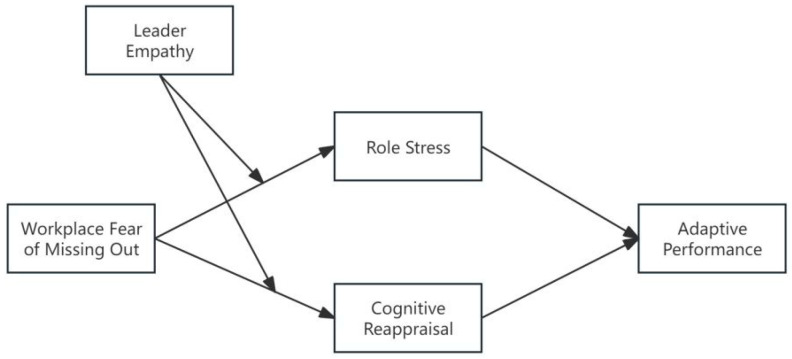
Conceptual model used in this research.

Based on the aforementioned analysis, this study has constructed a theoretical model, as illustrated in Figure 1.

## 3. Research Design

### 3.1. Research Procedure and Sample

This study followed five sequential methodological phases, which are summarized below: 

Phase 1: Literature review and hypothesis development

↓

Phase 2: Instrument development

↓

Phase 3: Pilot study (n = 30)

↓

Phase 4: Main survey (N = 510; valid n = 442)

↓

Phase 5: Data analysis (CFA + hierarchical regression + fsQCA)

This study employed a cross-sectional survey design. Data were collected between September 2022 and October 2024. New employees (tenure < 3 years) from enterprises in eastern and central China were recruited using convenience and snowball sampling. Because newly employed workers were relatively difficult to access through formal organizational channels, probability sampling was not feasible in the present study, and snowball sampling further facilitated participant recruitment through peer referrals. The inclusion criteria were full-time status, direct supervisor relationship, and voluntary participation. The exclusion criteria were part-time/temporary status, internship without contract, organizations with fewer than 10 employees, or excessive missing data. A pilot study with 30 new employees confirmed item clarity and scale reliability (Cronbach’s α > 0.70) prior to the main survey.

A total of 510 questionnaires were distributed (350 online, 160 offline). After excluding 68 invalid questionnaires (e.g., straight-lining, excessive completion time > 1000 s, incomplete responses), 442 valid responses were retained (effective rate: 86.7%). Among the valid sample, 50.7% were male, 45.2% were aged 21–25 years, and 51.8% were aged 26–30 years. Overall, 79.0% held a bachelor’s degree, and respondents were primarily from private enterprises (55.9%) and state-owned enterprises (27.8%). All participating organizations were adopting AI-based tools (e.g., intelligent decision support systems, generative AI), ensuring that respondents’ workplace experiences reflected an AI-driven context.

### 3.2. Variable Measurement

This study includes five core variables: WFMO, role stress, cognitive reappraisal, adaptive performance, and leader empathy. All questionnaires were administered in Chinese. The complete questionnaire is shown in Appendix A. Three of the scales were translated using standard translation-back-translation procedures, while the other two had already been validated in Chinese in prior studies (Wang et al., 2007; X. Y. Liu & Wan, 2016). All measurement scales employed in this study have been widely validated in prior domestic and international research, demonstrating sound psychometric properties. Specifically, WFMO, role stress, adaptive performance, and leader empathy were measured using a 5-point Likert scale ranging from 1 (“strongly disagree”) to 5 (“strongly agree”). These constructs are relatively concrete and intuitive, and the 5-point scale effectively captures respondents’ general attitudes and perceptions while reducing cognitive burden. Cognitive reappraisal was assessed using a 7-point Likert scale ranging from 1 (“strongly disagree”) to 7 (“strongly agree”). This scale was derived from the Emotion Regulation Questionnaire developed by Gross and subsequently revised by Wang Li and colleagues, who divided emotion regulation into two dimensions: Cognitive reappraisal was measured using the 6-item scale revised by Wang et al. (2007). As a relatively complex emotion regulation strategy involving subtle cognitive and emotional processes, cognitive reappraisal benefits from a wider response range. The 7-point scale allows for finer discrimination of respondents’ attitudes, thereby improving measurement precision. Previous studies have extensively employed this scale, confirming its high reliability and validity in capturing complex psychological constructs.

WFMO. This variable was measured using the 10-item scale developed by Budnick et al. A sample item is “I worry that I will miss important work updates.” In the present study, the scale demonstrated high internal consistency, with a Cronbach’s α of 0.895. The scale items (e.g., “I worry that I will miss important work updates”) inherently capture concerns amplified by AI-accelerated information flows and algorithmic performance tracking.

Role stress. Role stress was assessed using the 13-item scale developed by Peterson, which comprises three dimensions: role overload, role conflict, and role ambiguity (Peterson et al., 1995). Sample items include “There are times when I need to reduce certain aspects of my role,” “I often encounter situations where conflicting demands arise,” and “I have clear goals and objectives for my work.” The Cronbach’s α for this scale in the current study was 0.871.

Cognitive reappraisal. Cognitive reappraisal was measured using the 6-item scale revised by Wang et al. (2007). A representative item is “When I want to feel more positive emotions (such as happiness or pleasure), I change my way of thinking about the situation.” The scale exhibited good reliability in this study, with a Cronbach’s α of 0.851.

Adaptive performance. Adaptive performance was measured using the 9-item scale developed by Griffin et al. (2007). A sample item is “I am able to perform the core aspects of my job well.” The Cronbach’s α for this scale was 0.859.

Leader empathy. Leader empathy was assessed using a 16-item scale adapted to the Chinese organizational context by (X. Y. Liu & Wan, 2016). A sample item is “My leader can accurately perceive my low mood.” This scale demonstrated excellent internal consistency in the present study, with a Cronbach’s α of 0.939.

Control variables. Gender, age, education level, work experience, and organizational affiliation were included as controls based on established relationships with newcomer adaptation (H. He & Huang, 2015). Organizational affiliation was coded as: 1 = state-owned enterprise, 2 = private enterprise, 3 = wholly foreign-owned enterprise, 4 = Sino-foreign joint venture, 5 = public institution. This variable was treated as a categorical control in regression analyses.”

The use of different Likert scales (5-point vs. 7-point) was based on the nature of the constructs. More concrete constructs (WFMO, role stress, adaptive performance, leader empathy) were measured with a 5-point scale to reduce cognitive burden, whereas cognitive reappraisal—a more complex emotion regulation strategy—was measured with a 7-point scale to allow finer discrimination, following prior validation (Wang et al., 2007). Potential response bias from mixing scale formats was assessed via Harman’s single-factor test (P. M. Podsakoff et al., 2003), which indicated no serious common method bias.

### 3.3. Statistical Analysis

This study employed SPSS 27.0 for descriptive statistics, correlation analysis, and hierarchical regression analysis for hypothesis testing. AMOS 28.0 was used exclusively for confirmatory factor analysis to assess construct validity. fsQCA 3.0 software was applied for configurational analysis. The analytical procedures were conducted as follows: (1) testing for common method bias using SPSS 27.0 and AMOS 28.0 (P. M. Podsakoff & Organ, 1986; P. M. Podsakoff et al., 2003); (2) conducting confirmatory factor analysis with AMOS 28.0; (3) performing descriptive statistics and correlation analyses using SPSS 27.0; (4) testing research hypotheses using SPSS 27.0; and (5) implementing fuzzy set qualitative comparative analysis using fsQCA software (Ragin, 2008; Ragin & Davey, 2016).

## 4. Data Analysis and Hypothesis Testing

### 4.1. Common Method Bias Test

As the data in this study were collected from self-reported questionnaires and all variables were measured at a single time point, the potential risk of common method bias cannot be fully excluded. To mitigate the influence of social desirability and subjective response tendencies, an anonymous survey procedure was adopted during data collection to reduce respondents’ evaluation concerns. In addition, Harman’s single-factor test was conducted (P. M. Podsakoff & Organ, 1986). The results showed that 33 factors with eigenvalues greater than 1 were extracted, and the first factor accounted for only 17.198% of the total variance, which is below the recommended threshold of 40% (P. M. Podsakoff et al., 2003). These findings suggest that common method bias was unlikely to pose a serious threat to the validity of the study.

### 4.2. Confirmatory Factor Analysis

Confirmatory factor analysis (CFA) was conducted using AMOS 28.0 to examine the structural validity of the five latent variables: WFMO, role stress, cognitive reappraisal, adaptive performance, and leader empathy. Given the relatively large number of measurement items, item parceling was employed to improve model parsimony and estimation stability (F. Wei et al., 2024). Following the recommendations of Rogers and Schmitt (2004), a balanced parceling strategy was adopted, with each latent construct represented by three parcels. CFA was then performed to evaluate the overall model fit and discriminant validity among the constructs (Rogers & Schmitt, 2004).

The following fit indices were used: χ^2^/df (chi-square to degrees of freedom ratio), RMSEA (root mean square error of approximation), SRMR (standardized root mean square residual), IFI (incremental fit index), CFI (comparative fit index), and TLI (Tucker–Lewis index). The CFA results are presented in Table 1. The five-factor model demonstrated good fit to the data (χ^2^/df = 2.856, RMSEA = 0.065, SRMR = 0.059, IFI = 0.925, CFI = 0.924, TLI = 0.900), all meeting the recommended cutoff criteria. These results indicate satisfactory discriminant validity among the five constructs and provide a solid empirical foundation for subsequent statistical analyses.

It should be noted that AMOS was used exclusively for confirmatory factor analysis to assess construct validity; hypothesis testing was conducted using hierarchical regression in SPSS.

### 4.3. Descriptive Statistics and Correlation Analysis

This study utilized SPSS software to measure the mean (M), standard deviation (SD), and correlation among WFMO, role stress, cognitive reappraisal, adaptive performance, leader empathy ability, and control variables. The specific results are presented in the table. According to Table 2, there is a significant positive correlation between WFMO and role stress among new employees (r = 0.644, *p* < 0.01), as well as between WFMO and cognitive reappraisal (r = 0.468, *p* < 0.01), thus providing preliminary support for H2 and H5. Additionally, there is a significant positive correlation between WFMO and adaptive performance among new employees (r = 0.437, *p* < 0.01), providing preliminary support for H1. The correlation analysis results align with theoretical expectations, and the relevant hypotheses proposed in this study have been preliminarily confirmed.

### 4.4. Hypothesis Testing

This study employed AMOS 28.0 and SPSS 27.0 to conduct hypothesis testing using hierarchical regression analysis. Prior to analysis, multicollinearity was assessed, and the variance inflation factor (VIF) values for all models were below 3, indicating that multicollinearity was not a concern. To test mediation, we followed Baron and Kenny’s (1986) four-step procedure: (a) the independent variable (WFMO) must significantly predict the dependent variable (adaptive performance); (b) WFMO must significantly predict each mediator (role stress and cognitive reappraisal); (c) each mediator must significantly predict adaptive performance when controlling for WFMO; and (d) the direct effect of WFMO on adaptive performance should be reduced after including the mediator. In addition, to reduce potential estimation bias arising from interaction terms, all relevant variables were mean-centered before analysis.

#### 4.4.1. Direct and Mediating Effect Test

The results of the direct effect analysis are reported in Models 1, 2, and 3 in Table 3. Model 1 serves as the baseline model, while Model 2 incorporates WFMO. As shown in Table 3, WFMO has a significant positive effect on new employees’ adaptive performance (β = 0.326, *p* < 0.001).

The mediation effect analysis results are also presented in Table 3. WFMO is significantly and positively associated with adaptive performance (β = 0.326, *p* < 0.001), supporting H1. Models 9 and 11 indicate that WFMO has significant positive effects on role stress (β = 0.512, *p* < 0.001) and cognitive reappraisal (β = 0.508, *p* < 0.001), satisfying the first condition for mediation testing. Furthermore, Models 3 and 5 demonstrate that role stress (β = 0.508, *p* < 0.001) and cognitive reappraisal (β = 0.424, *p* < 0.001) each exert significant positive effects on adaptive performance, fulfilling the second condition for mediation.

As shown in Models 4 and 6, when role stress and cognitive reappraisal are entered into the regression models, the positive effect of WFMO on adaptive performance is attenuated. Specifically, after including role stress, the coefficient of WFMO decreases from 0.326 (*p* < 0.001) to 0.105 (*p* < 0.001); similarly, after including cognitive reappraisal, the coefficient decreases to 0.137 (*p* < 0.001). These results satisfy the third condition for mediation and provide empirical support for Hypotheses 2 and 3 (H2 and H3). To further enhance the robustness of the findings, the mediating effects of role stress and cognitive reappraisal were additionally tested using the PROCESS macro in SPSS, and the results consistently confirmed H2 and H3.

This study further examined the mediating effect using the BOOTSTRAP method. As shown in Table 4, the indirect effect of WFMO on adaptive performance through role stress was 0.147, with a 95% CI of 0.090, 0.207, which does not include 0; the indirect effect of WFMO on adaptive performance through cognitive reappraisal was 0.161, with a 95% CI of 0.117, 0.212, which does not include 0. Therefore, H2 and H3 were further verified. To test the mediating effects, we employed the bootstrapping procedure with 5000 resamples. Compared with traditional causal steps approaches (e.g., the Sobel test), bootstrapping does not assume multivariate normality of the sampling distribution of the indirect effect and therefore provides greater statistical power and more accurate confidence intervals for indirect effects (MacKinnon et al., 2004; Preacher & Hayes, 2008). As shown in Table 5, the 95% bias-corrected bootstrap confidence intervals for the indirect effects of role stress [0.090, 0.207] and cognitive reappraisal [0.117, 0.212] both excluded zero, confirming that both mediating paths were statistically significant.

#### 4.4.2. Moderation Effect Test

To examine the moderating role of leaders’ empathy ability in the relationship between WFMO and role stress, Hypothesis 4a (H4a) was proposed, suggesting that leaders’ empathy ability moderates the association between WFMO and role stress among new employees. Specifically, higher levels of leaders’ empathy ability were expected to strengthen, whereas lower levels were expected to weaken, the relationship between WFMO and role stress. Similarly, to investigate the moderating effect of leaders’ empathy ability on the relationship between WFMO and cognitive reappraisal, Hypothesis 4b (H4b) was proposed, positing that leaders’ empathy ability moderates the association between WFMO and cognitive reappraisal among new employees, such that the relationship is stronger under conditions of high leaders’ empathy ability and weaker under conditions of low leaders’ empathy ability.

**Figure 2:**
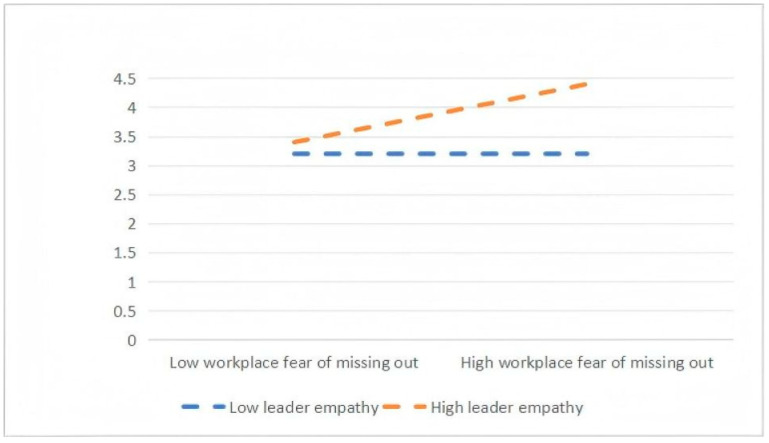
Simple slope analysis of workplace fear of missing out on role stress at low and high levels of leader empathy (M ± 1 SD).

**Figure 3:**
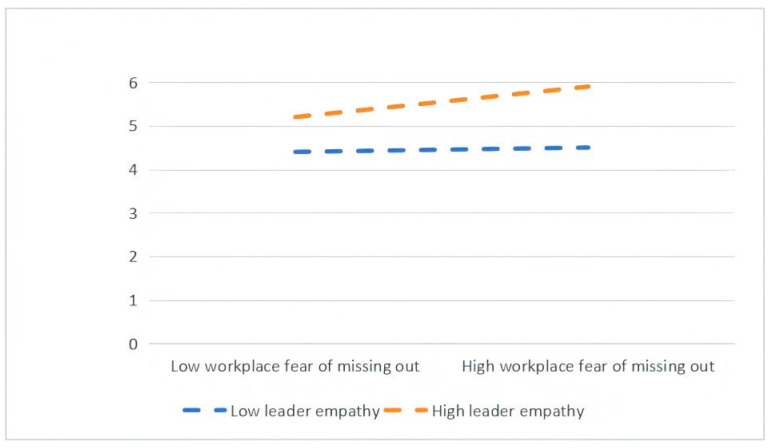
Simple slope analysis of workplace fear of missing out on cognitive reappraisal at low and high levels of leader empathy (M ± 1 SD).

To test these hypotheses, a three-step hierarchical regression analysis was conducted. Interaction terms between WFMO and leaders’ empathy ability were introduced in the final step to examine the presence of moderation effects. A significant increase in explained variance (ΔR^2^) after adding the interaction terms was taken as evidence of a moderating effect. As shown in Table 5, the interaction between WFMO and leaders’ empathy ability has a significant positive effect on both role stress (β = 0.260, *p* < 0.001) and cognitive reappraisal (β = 0.140, *p* < 0.001), providing empirical support for H4a and H4b. Furthermore, as illustrated in Figure 2, the positive relationship between WFMO and role stress is stronger when leaders’ empathy ability is high, further supporting H4a. Likewise, Figure 3 shows that under conditions of high leaders’ empathy ability, the positive effect of WFMO on cognitive reappraisal is amplified, thereby offering additional support for H4b. “Simple slopes were plotted at ±1 standard deviation from the mean of leader empathy.”

#### 4.4.3. Moderated Mediation Effect

The results of the moderated mediation effect test are presented in Table 6. When new employees are in an environment with high leader empathy, the indirect effect of role stress is significant (β = 0.113, 95% CI 0.045, 0.181); when new employees are in an environment with low leader empathy, the indirect effect of role stress is not significant (β = 0.021, 95% CI −0.0002, 0.05), confirming H5a. Furthermore, when new employees are in an environment with high leader empathy, the indirect effect of cognitive reappraisal is significant (β = 0.065, 95% CI 0.037, 0.099); when new employees are in an environment with low leader empathy, the indirect effect of cognitive reappraisal is not significant (β = 0.017, 95% CI -0.01, 0.053), confirming H5b.

## 5. Qualitative Comparative Analysis of Fuzzy Sets

Fuzzy set qualitative comparative analysis (fsQCA) is a configurational analytical approach grounded in fuzzy set theory and is particularly suitable for examining complex causal relationships. Unlike traditional statistical methods that emphasize net effects of individual variables, fsQCA focuses on how different configurations of antecedent conditions jointly influence outcome variables, thereby capturing causal asymmetry and equifinality. This method enables the identification of multiple causal pathways leading to the same outcome rather than isolating a single linear relationship, making it especially appropriate for research contexts characterized by complexity and causal plurality (M. Zhang & Du, 2019). Prior studies have noted that fsQCA can effectively complement regression-based analyses by uncovering complex causal combinations that are difficult to detect using conventional quantitative techniques alone, thus providing a more comprehensive analytical perspective (Fiss et al., 2013).

The primary objective of this study is to explore how different combinations of antecedent conditions are associated with new employees’ adaptive performance, rather than to examine the isolated effect of a single predictor. This research focus aligns closely with the methodological strengths of fsQCA. Specifically, new employees’ adaptive performance may emerge from the joint action of multiple antecedent factors, and the effective combinations of these conditions may differ across individuals. In such cases, traditional statistical approaches often struggle to capture the diversity and nonlinearity of causal configurations (M. Zhang & Du, 2019). By contrast, fsQCA allows for the identification of shared causal mechanisms across multiple pathways by systematically analyzing condition configurations, thereby offering a deeper and more nuanced explanation of the underlying causal logic. Accordingly, this study introduces fsQCA following hierarchical regression analysis to compensate for the limited explanatory capacity of purely quantitative methods in addressing complex causal relationships. Through this approach, the influence pathways formed by different combinations of factors on new employees’ adaptive performance can be more clearly identified, enhancing both the explanatory depth and robustness of the research findings.

### 5.1. Variable Calibration

The conditions were selected and constructed as follows. Seven conditions were selected based on the theoretical model and empirical hypotheses: workplace fear of missing out, role stress, cognitive reappraisal, leader empathy, adaptive performance (outcome), age, and unit nature. These conditions represent the core variables in the hypothesized relationships. For fsQCA, each condition is treated as a set in which cases have varying degrees of membership. The * symbol denotes logical AND (intersection), indicating that all listed conditions must be present simultaneously. The ~ symbol denotes logical NOT (negation), indicating the absence of a condition. For example, ~WFMO represents low or absent workplace fear of missing out. fsQCA requires calibrated set membership scores and asymmetric causality logic. The continuous survey data were calibrated using three anchors (full membership, crossover point, full non-membership), and the sample size (n = 442) exceeds the recommended minimum for robust configurational analysis.

Prior to conducting the qualitative comparative analysis, all variables were calibrated to ensure their suitability for fsQCA. In this study, the mean values of seven variables—WFMO, role stress, cognitive reappraisal, leader empathy ability, adaptive performance, age, and unit type—were calculated for calibration purposes. Following the membership criteria proposed by Ragin, the 95th percentile, 5th percentile, and median (50th percentile) of the original data were used as the anchors for full membership, full non-membership, and the crossover point, respectively (Ragin, 2009). The specific calibration thresholds are presented in Table 7.

For the age variable, a direct calibration approach was adopted: respondents aged 20 years and below were assigned a membership score of 0, those aged 21–25 were assigned 0.33, those aged 26–30 were assigned 0.67, and those aged 31 years and above were assigned a score of 1. Regarding unit type, the categories were coded from 1 to 5, representing state-owned enterprises, private enterprises, wholly foreign-owned enterprises, Sino-foreign joint ventures, and public institutions, respectively. A direct calibration method was applied, with 5 set as the full membership point, 3.5 as the crossover point, and 1 as the full non-membership point (Jacobs & Cambré, 2020). To avoid the exclusion of cases with a membership score of exactly 0.5 during the fsQCA analysis, values of 0.5 were adjusted to 0.499 in accordance with prior methodological recommendations (Campbell et al., 2016).

### 5.2. Univariate Necessity Analysis

This study further conducted a univariate necessity analysis on each variable, aiming to evaluate the independent impact of each independent variable on the outcome variable. If the consistency is greater than 0.8, X is a sufficient condition for Y; if the consistency is greater than 0.9, X is considered a necessary condition for Y (Fiss, 2011). The results are shown in Table 8. The consistency rates of each individual variable affecting adaptive performance did not reach the standard value of 0.9 for a necessary condition, indicating that a single antecedent variable cannot explain the mechanism by which new employees’ adaptive performance is affected. The explanatory power of the results is weak and does not constitute a necessary condition. Therefore, it is necessary to analyze adaptive performance in combination with multiple antecedent conditions.

### 5.3. Analysis of Condition Configuration Sufficiency

Firstly, a truth table was constructed, and through logical operations in Boolean algebra,47 and referencing the research of Greckhamer, this study had 442 valid samples, with a large sample size (Greckhamer et al., 2013). To avoid configurations with excessively low frequencies, the minimum case frequency was set at 3, retaining 90% of the original cases, meeting the requirement of retaining about 80% of the original cases (Verbeke et al., 2019; Douglas et al., 2020). Secondly, during the analysis, the consistency threshold was set at 0.8 to ensure the explanatory power of the configurations (Leppänen et al., 2019). Finally, the PRI consistency threshold was set at 0.7 to avoid the issue of “simultaneous subset relationships” (Dwivedi et al., 2018). The combination of two antecedent configurations is shown in Table 9. The overall consistency coefficient of the conditional configurations was 0.940, and the overall coverage rate was 0.585, indicating that the two configurations covered 58.5% of the adaptive performance samples.

#### 5.3.1. Configuration 1: High Anxiety–High Stress-Driven Type

Configuration 1 indicates that the antecedent configuration co-occurring with adaptive performance is Workplace Fear of Missing Out * role stress * cognitive reappraisal * leader empathy ability. In this configuration, WFMO, role stress, cognitive reappraisal, and leader empathy ability all function as core conditions for the generation of high adaptive performance. The results suggest that when new employees simultaneously experience high levels of WFMO and role stress, and when these experiences are accompanied by a high level of cognitive reappraisal as well as strong empathetic support from leaders, adaptive performance is more likely to emerge. From the perspective of conservation of resources theory, individuals tend to increase psychological and behavioral resource investment when facing adaptation demands in a new environment. Configuration 1 (P1): High WFMO * High Role Stress * High Cognitive Reappraisal * High Leader Empathy. Original coverage: 0.558; unique coverage: 0.395; consistency: 0.941. This configuration indicates that WFMO, role stress, cognitive reappraisal, and leader empathy function as core conditions for high adaptive performance. Detailed theoretical interpretation is provided in Section 6.1.

#### 5.3.2. Configuration 2: Low Anxiety–Low Stress Compensation Type

Configuration 2 (P2): Age * Unit Nature * ~WFMO * ~Role Stress * High Cognitive Reappraisal * High Leader Empathy. Original coverage: 0.189; unique coverage: 0.027; consistency: 0.945. This configuration indicates that age, unit nature, cognitive reappraisal, and leader empathy emerge as core conditions. Detailed theoretical interpretation is provided in Section 6.1.

## 6. Conclusions and Discussion

### 6.1. Research Conclusions

Note on causal language: this study reveals a dual-path driving mechanism through which WFMO is positively associated with new employees’ adaptive performance in the context of AI-driven technological iteration. The underlying effect reflects a dynamic compensatory process under conditions of perceived resource vulnerability. Drawing on an integrated framework of conservation of resources theory (Hobfoll, 1989; Halbesleben et al., 2014) and the emotion regulation process model (Gross, 1998), the following conclusions are obtained.

WFMO among new employees has a significant positive effect on adaptive performance. WFMO primarily manifests as concern over potential losses of multiple resources, including information access, career opportunities, technological relevance, and social connections. The findings indicate that, when confronted with such anxiety, new employees tend to proactively adjust their resource allocation strategies. This form of “defensive resource mobilization” challenges the traditional assumption that anxiety is uniformly detrimental (Cheng & McCarthy, 2018; Przybylski et al., 2013) and supports the conservation of resources principle that loss prevention is prioritized over resource acquisition (Hobfoll, 1989). Moreover, in AI-enabled work environments characterized by rapid technological updates and abundant learning opportunities (Song et al., 2021), the potential negative impact of anxiety is partially buffered. As a result, new employees are more likely to interpret WFMO as a developmental signal that motivates growth-oriented behavior, consistent with recent findings that moderate anxiety can stimulate proactive adaptation (Budnick et al., 2020; Gartner et al., 2022).

Role stress and cognitive reappraisal statistically mediate this relationship between WFMO and adaptive performance. The dual mediating paths of role stress and cognitive reappraisal illuminate the micro-level mechanism through which anxiety-related emotional energy is transformed. On the one hand, role stress induces a sense of urgency that encourages new employees to accelerate skill acquisition and role adaptation, thereby forming a positive feedback loop from stress to capability development (LePine et al., 2004; N. P. Podsakoff et al., 2007). On the other hand, cognitive reappraisal enables employees to reinterpret WFMO as an indicator of professional development opportunities through meaning reconstruction (e.g., viewing mastery of AI tools such as ChatGPT as a means of enhancing competitiveness). This process facilitates the directional transformation of emotional arousal into goal-directed behavior, consistent with the cognitive modulation logic embedded in the emotion regulation process model (Gross, 1998; Goldin et al., 2009). In this regard, conservation of resources theory explains why resources are mobilized and transformed (Hobfoll, 1989), whereas the emotion regulation process model explains how this transformation occurs (Gross, 1998). This dual-path framework extends prior research that has predominantly examined anxiety effects through a single theoretical lens (Cheng & McCarthy, 2018).

Leaders’ empathy positively moderates the relationships between WFMO and role stress, as well as between WFMO and cognitive reappraisal. In contexts characterized by high leader empathy, WFMO is more likely to elicit heightened role stress and increased engagement in cognitive reappraisal, thereby contributing to higher adaptive performance. Empathetic leaders can attenuate the negative emotional experiences associated with WFMO through accurate emotional perception and supportive behaviors, enhancing employees’ psychological resilience under stress. For example, in technology-intensive organizations, empathetic leaders may help employees convert anxiety into learning motivation through emotional support and targeted resource provision, whereas in more traditional organizational settings, leader empathy may function by fostering a supportive interpersonal climate that mitigates the inhibiting effects of anxiety (X. Y. Liu & Wan, 2016). This finding is consistent with prior research demonstrating that empathetic leadership serves as both emotional and instrumental resources for employee adaptation.

Two distinct configurational paths lead to high adaptive performance among new employees. This study reveals two formation paths of new employees’ adaptive performance, namely: Path P1 “WFMO * role stress * cognitive reappraisal * leader empathy ability”; Path P2 “age * unit nature * ~ WFMO * ~ role stress * cognitive reappraisal * leader empathy ability”. Among them, the coverage rate of P1 is significantly higher than that of P2, indicating that the explanatory power of P1 is stronger; that is, the combined effect of WFMO, role stress, cognitive reappraisal, and leader empathy ability can explain more cases of new employees’ adaptive performance. Therefore, the overall model hypothesis proposed in this study has been further validated, emphasizing the importance of the interaction between psychological and social factors in the formation process of new employees’ adaptive performance (Fiss et al., 2013; M. Zhang & Du, 2019).

Configurational paths to adaptive performance. The fsQCA results reveal two distinct pathways to high adaptive performance, underscoring the equifinal nature of newcomer adaptation (Fiss et al., 2013; Ragin, 2009).

Path 1: High Anxiety–High Stress-Driven Type. When new employees simultaneously experience high levels of WFMO and role stress, and when these experiences are accompanied by a high level of cognitive reappraisal as well as strong empathetic support from leaders, adaptive performance is more likely to emerge. From the perspective of conservation of resources theory, individuals tend to increase psychological and behavioral resource investment when facing adaptation demands in a new environment (Hobfoll, 1989; Halbesleben et al., 2014). Meanwhile, according to the emotion regulation process model, new employees confronted with WFMO—a salient negative emotional experience during early organizational entry—are likely to activate emotion regulation strategies to manage their affective responses (Gross, 1998). This configuration is consistent with the hypotheses proposed in this study. Moreover, compared with other configurations, this path demonstrates relatively high unique coverage, indicating that WFMO and role stress play central roles in shaping adaptive performance. This finding highlights the importance of psychological factors in new employees’ adaptation processes, suggesting that organizations should pay close attention to employees’ emotional and stress-related experiences during early socialization. From a managerial perspective, this configuration underscores the necessity of strengthening systematic support mechanisms for new employee adaptation, including mental health monitoring and intervention systems, as well as incorporating leaders’ empathy ability into management development programs.

Path 2: Low Anxiety–Low Stress Compensation Type. Relatively older new employees, when supported by strong leader empathy and effective cognitive reappraisal strategies, are more likely to achieve high adaptive performance, even in the presence of WFMO. According to the emotion regulation process model, new employees entering an unfamiliar work environment tend to regulate negative emotions such as WFMO through cognitive strategies that reshape emotional appraisals (Gross, 1998). From the perspective of conservation of resources theory, this configuration highlights the role of individual psychological resources and contextual conditions in enabling employees to protect and mobilize resources under stress (Hobfoll, 1989). This path further reveals that both individual characteristics (e.g., age) and organizational characteristics (e.g., unit nature) jointly influence adaptive performance, indicating that employees with different backgrounds may require differentiated support mechanisms. From a management standpoint, this configuration suggests that organizations should adopt flexible and differentiated onboarding and support strategies. For example, older new employees may benefit from greater autonomy and reduced procedural constraints, allowing them to draw on prior experience to adapt more effectively (Bakker & Demerouti, 2017). Similarly, onboarding practices should be tailored to the specific characteristics of different organizational contexts, ensuring that support resources align with organizational culture and environmental demands.

### 6.2. Research Contributions

Firstly, this study provides an innovative explanatory framework for understanding adaptive performance among early-career employees in the context of AI-driven work environments. Existing research has predominantly focused on experienced or general employee groups (Pulakos et al., 2000; Griffin et al., 2007), whereas this study highlights the stage-specific characteristics of WFMO among new employees. Specifically, a dynamic tension emerges between resource vulnerability induced by role ambiguity and cognitive agency activated by career development demands (Bakker & Demerouti, 2017; Grant & Ashford, 2008). Prior research indicates that younger employees are more inclined to cope with uncertainty by increasing effort investment, thereby enhancing career competitiveness (Morrison & Phelps, 1999). Building on this psychological profile, the present study demonstrates that WFMO can be transformed from an emotional experience into adaptive behavior through a dual-path mechanism. This finding challenges the traditional research paradigm that conceptualizes WFMO solely as a negative state (Halbesleben et al., 2014; Elhai et al., 2020a). Theoretically, by integrating conservation of resources theory with the emotion regulation process model, this study proposes a dual-path explanatory framework centered on resource vulnerability and cognitive agency. This integration not only extends the applicability of conservation of resources theory to early-career employees but also enriches the emotion regulation process model by revealing how cognitive strategies compensate for perceived resource deficits in organizational contexts.

Secondly, this study disentangles the dual driving effects of WFMO and establishes the moderating role of leaders’ empathy ability. Unlike prior research that explains anxiety effects in a unidirectional manner (Budnick et al., 2020; Shi et al., 2023), this study demonstrates that WFMO simultaneously influences adaptive performance through role stress and cognitive reappraisal, offering an integrated explanation for the divergence between “anxiety depletion theory” and “anxiety drive theory” (Cheng & McCarthy, 2018; Przybylski et al., 2013). This contribution advances theory in three key respects. First, by empirically distinguishing resource mobilization and resource management processes, the study confirms the complementarity rather than competition between conservation of resources theory and the emotion regulation process model. Second, it enriches the situational dimension of the emotion regulation process model by validating the activating role of external emotional resources—namely leaders’ empathy—on internal emotion regulation strategies, thereby extending the model to incorporate social-contextual regulation mechanisms. Third, by revealing asymmetrical regulatory effects across dual paths, the study demonstrates variability in how external emotional resources facilitate internal resource transformation, establishing a multi-channel gain model of anxiety-driven behavior.

Thirdly, this study advances the integrated application of multiple methods to uncover the configurational mechanisms underlying new employees’ adaptive performance. Addressing the limitations of single-method approaches, this study follows the methodological logic advocated by Fiss et al. (2013) by complementing regression analysis with fuzzy set qualitative comparative analysis (Ragin, 2009; Fiss et al., 2013). This integration provides a novel analytical perspective for capturing the complexity and multiplicity of causal mechanisms. The findings identify two distinct configurational paths to adaptive performance: a “high anxiety–high stress-driven” path and a “low anxiety–low stress compensation” path. In the former, WFMO and role stress function as core triggering conditions, whereas in the latter, organizational support and individual characteristics play dominant roles. This finding extends leadership theory from a single behavioral perspective to a multi-level “situation–cognition–behavior” framework.

### 6.3. Management Implications

Based on the empirical findings that WFMO is positively associated with adaptive performance through dual pathways of role stress and cognitive reappraisal, and that leader empathy amplifies both pathways, the following practical strategies are proposed. These implications are grounded in established organizational behavior literature while remaining aligned with the study’s core results.

Information transparency to manage WFMO. Given that WFMO stems from perceived information gaps and competitive pressure (Budnick et al., 2020; Shi et al., 2023), organizations should reduce ambiguity through structured information sharing. Regular team briefings, clear career development roadmaps, and accessible knowledge repositories can mitigate the uncertainty that drives excessive WFMO. Rather than eliminating WFMO entirely—which may inadvertently reduce the adaptive motivation it can generate—managers should channel it constructively by ensuring employees have timely access to relevant information and skill development opportunities.

Cognitive reappraisal training for newcomers. The finding that cognitive reappraisal mediates the WFMO–adaptive performance relationship suggests that training programs enhancing emotion regulation skills can improve newcomer adaptation (Gross, 1998; Wang et al., 2007). Organizations may incorporate brief cognitive reappraisal exercises into onboarding programs, such as guided reflection on anxiety-provoking situations and reframing techniques (Goldin et al., 2009). This approach is consistent with evidence that emotion regulation training enhances resilience in dynamic work environments (Bakker & Demerouti, 2017).

Leader empathy development. As leader empathy emerged as a significant moderator, organizations should invest in empathy training for supervisors of new employees (X. Y. Liu & Wan, 2016). Effective interventions include perspective-taking exercises, active listening workshops, and feedback mechanisms that enhance leaders’ sensitivity to newcomer anxiety. Importantly, the findings suggest that empathetic leadership does not merely reduce stress but amplifies the adaptive potential of moderate WFMO by facilitating both resource mobilization and cognitive reframing.

Tailored support based on anxiety profiles. The fsQCA results, revealing two distinct configurational paths, suggest that uniform onboarding approaches may be suboptimal. For newcomers exhibiting high WFMO and high role stress (Path 1), intensive leader support and structured skill development are critical. For those with lower WFMO but strong cognitive reappraisal capacity (Path 2), autonomy and resource provision may suffice (Fiss et al., 2013; M. Zhang & Du, 2019). This differentiated approach aligns with person–environment fit theory and conservation of resources principles (Hobfoll, 1989).

### 6.4. Limitations

Although this study has yielded meaningful findings, several limitations should be acknowledged. First, this study assumed a linear positive effect of WFMO, but very high levels may be detrimental; future research should explore curvilinear (inverted U-shaped) relationships. The cross-sectional design precludes causal inference; observed associations may reflect reverse causality or unmeasured confounding. Longitudinal designs are needed to establish temporal precedence.

First, the stage delineation of new employees warrants further refinement. This study defines new employees as those within three years of organizational entry, which broadly captures the main phases of organizational socialization; however, this definition differs from the more restrictive “first 90 days” criterion commonly used in prior research (H. He & Huang, 2015) and may obscure heterogeneity across critical adaptation stages. Future studies could integrate career stage development theory and adopt a phased longitudinal design, using milestones such as six months, one year, and three years after entry to examine the dynamic evolution of WFMO and to construct stage-specific adaptive intervention models.

Second, the use of cross-sectional data limits the ability to capture reciprocal effects and lagged relationships between WFMO and adaptive performance. Future research may employ longitudinal or experience sampling designs with multi-wave data collection at key time points (e.g., quarterly or annual assessments) to reveal dynamic feedback mechanisms between anxiety and adaptive behavior. In addition, incorporating neurophysiological indicators to objectively assess anxiety-related arousal could help mitigate common method bias inherent in self-report measures and facilitate a shift toward multimodal data integration in organizational behavior research.

Third, this study employed a mixed-mode data collection approach combining online and offline surveys, and participants were recruited using convenience sampling and snowball sampling, which may introduce mode effects and limit the generalizability of the findings. Prior research suggests that online and offline surveys may differ in terms of response rates, response styles, and data quality (Deutskens et al., 2006; Markovitch & Yoo, 2009). Specifically, online respondents may exhibit higher survey fatigue and lower attention levels compared to offline respondents, potentially affecting the reliability of self-reported measures. Although the present study found no significant differences in response patterns between the two modes, the potential for mode-related bias cannot be fully excluded. Future research should consider employing unified data collection methods or statistically controlling for mode effects to enhance data comparability.

Fourth, this study does not explicitly account for the moderating role of industry characteristics. For instance, employees in high-technology sectors may experience stronger negative effects of WFMO due to skill obsolescence pressure (Scheck & Kinicki, 2000), whereas greater autonomy in creative industries may enhance the functional role of cognitive reappraisal. Empirical evidence suggests that industry context significantly influences leadership effectiveness and employee adaptation outcomes. For example, empathetic leadership may be more critical in service-oriented industries where emotional labor is prevalent, while in manufacturing sectors, instrumental support may carry greater weight. Future research could conduct cross-industry comparative analyses by integrating macro-level indicators such as industry digitalization intensity and environmental uncertainty to examine contextual boundary conditions.

Fifth, although the AI-driven workplace context serves as the theoretical backdrop for this study, the role of AI was not directly measured as a construct. Our survey items assessed general perceptions of WFMO, role stress, cognitive reappraisal, and adaptive performance without explicitly referencing AI technologies. While all participating organizations were in the process of adopting AI-based tools, the extent to which individual respondents directly experienced or interacted with AI systems may vary. Consequently, the current study treats AI primarily as a contextual condition rather than an empirically operationalized variable. Future research should develop explicit measures of AI exposure and AI-related task demands to more rigorously test how specific AI features (e.g., algorithmic transparency, real-time performance feedback) shape newcomers’ psychological adaptation processes.

Sixth, the two-year data collection period (September 2022 to October 2024) coincided with the rapid proliferation of generative AI. We did not statistically control for the exact date of each response, leaving open the possibility that later respondents were differentially exposed to AI advancements. This temporal factor should be considered when interpreting the results, and future studies should either shorten the data collection window or include survey timing as a control variable.

Seventh, this study did not assess predictive validity through cross-validation or holdout sample techniques. Future research should employ split-sample validation to verify whether the observed relationships generalize to independent samples.

### 6.5. Future Research Directions

Building upon the findings and limitations of this study, several promising directions for future research are proposed. These directions not only address theoretical gaps but also have practical implications for organizational stakeholders.

First, future research should examine the differential effects of WFMO across industry sectors. As noted in the limitations, industry characteristics—such as technological intensity, competitive pressure, and regulatory environment—may significantly moderate the relationships identified in this study. For example, in high-technology sectors where skill obsolescence is rapid, WFMO may exert stronger effects on role stress and cognitive reappraisal, whereas in creative industries with higher autonomy, employees may more readily deploy cognitive reappraisal strategies. Cross-industry comparative studies would benefit organizational practitioners by identifying sector-specific intervention points. Human resource managers in technology-intensive firms could prioritize skill development programs and real-time feedback systems, while those in creative industries might focus on fostering autonomous work environments that facilitate cognitive flexibility.

Second, the dynamic evolution of WFMO over time warrants longitudinal investigation. The present study’s cross-sectional design precludes conclusions about causal directionality and temporal dynamics. Future research should adopt experience sampling methodology (ESM) or multi-wave panel designs to capture the fluctuation of WFMO, role stress, and cognitive reappraisal across critical adaptation periods (e.g., the first 90 days, six months, and one year). Such designs would enable researchers and practitioners to identify optimal intervention windows. For instance, if WFMO peaks during the first month and subsequently declines, organizations could front-load support resources during this critical period. Additionally, longitudinal data would facilitate the development of predictive models for early identification of employees at risk of maladaptive outcomes.

Third, the neurobiological underpinnings of WFMO and cognitive reappraisal represent an underexplored frontier. Integrating psychophysiological measures—such as cortisol levels, heart rate variability, and electroencephalography (EEG)—with self-report data could provide a more comprehensive understanding of the stress-regulation mechanisms underlying adaptive performance. This multi-method approach would benefit both researchers and organizational health practitioners by enabling objective assessment of intervention effectiveness. For example, biofeedback-based training programs could be developed to help new employees regulate their physiological responses to workplace stressors, complementing cognitive–behavioral strategies.

Fourth, the cross-cultural generalizability of the findings should be tested. The present study was conducted within the Chinese organizational context, where collectivist cultural values and hierarchical leadership norms may shape the experience and expression of WFMO differently than in individualist cultures. Cross-cultural replication studies would inform multinational corporations about the need for culturally adaptive onboarding and leadership development programs. For example, in high power-distance cultures, leader empathy may be perceived as particularly salient and expectation-raising, whereas in egalitarian cultures, peer support systems may play a more prominent role in facilitating newcomer adaptation.

Fifth, intervention studies are needed to translate the theoretical model into evidence-based practices. Experimental or quasi-experimental designs could test the efficacy of targeted interventions designed to enhance cognitive reappraisal skills or optimize leader empathy training. For example, randomized controlled trials could compare the effects of cognitive reappraisal training versus mindfulness-based stress reduction on new employees’ adaptive performance. Similarly, leader empathy interventions could be evaluated using pre-post designs with control groups. Such research would directly benefit organizational training and development departments by providing actionable, evidence-based guidelines for program design and implementation.

Finally, the integration of artificial intelligence tools in assessing and supporting new employee adaptation represents a promising avenue. AI-driven sentiment analysis of employee communications, predictive analytics for identifying at-risk newcomers, and personalized recommendation systems for learning and development resources could augment traditional human resource practices. Future research should examine the ethical implications, accuracy, and employee acceptance of such AI-augmented support systems, ensuring that technological solutions enhance rather than undermine the human-centric aspects of organizational socialization.

### 6.6. Conclusions

A caution regarding interpretation is necessary before concluding. Given the cross-sectional and non-experimental nature of the data, the following conclusions describe observed associations rather than established causal effects. This study makes several important contributions to understanding how WFMO relates to new employees’ adaptive performance in AI-driven work environments. By integrating conservation of resources theory and the emotion regulation process model, the research reveals a dual-path mechanism through which anxiety is transformed into adaptive behavior via role stress (resource mobilization pathway) and cognitive reappraisal (resource management pathway). The findings challenge the prevailing view of workplace anxiety as purely detrimental, demonstrating that moderate levels of WFMO can serve as a catalyst for proactive adaptation when properly channeled.

The study’s key findings can be summarized as follows: (1) WFMO is positively associated with new employees’ adaptive performance; (2) this relationship is mediated by both role stress and cognitive reappraisal, operating through distinct but complementary mechanisms; (3) leader empathy strengthens both mediating pathways, functioning as a critical contextual resource that amplifies the adaptive potential of anxiety; and (4) two distinct configurational paths—”high anxiety–high stress-driven” and “low anxiety–low stress compensation”—lead to high adaptive performance, underscoring the equifinal nature of newcomer adaptation.

From a practical standpoint, these findings suggest that organizations should adopt a nuanced approach to managing new employee anxiety rather than seeking to eliminate it entirely. By fostering leader empathy, providing cognitive reappraisal training, and creating supportive yet challenging environments, organizations can help newcomers transform anxiety into a motivational resource. The identification of multiple adaptation pathways also implies that one-size-fits-all onboarding programs may be suboptimal; instead, personalized interventions tailored to individual anxiety levels and resource profiles may yield superior outcomes.

In conclusion, this study advances theoretical understanding of newcomer adaptation in dynamic, technology-intensive work environments and provides actionable insights for organizational practice. Longitudinal or experimental designs are needed to confirm these patterns before strong causal claims can be made. As AI continues to reshape workplace demands, the ability to harness rather than suppress new employees’ anxiety about missing opportunities may become a critical determinant of both individual career success and organizational competitiveness.

## Figures and Tables

**Table 1:** Results of confirmatory factor analysis.

Model	Factor Structure	χ^2^/df	RMSEA	SRMR	IFI	CFI	TLI
Five-factor model	A, B, C, D, E	2.856	0.065	0.059	0.925	0.924	0.900
Four-factor model	A + B, C, D, E	3.235	0.064	0.102	0.770	0.769	0.758
Three-factor model	A + B + C, D, E	3.728	0.079	0.119	0.718	0.717	0.705
Two-factor model	A + B + C + D, E	4.123	0.082	0.115	0.677	0.675	0.662
One-factor model	A + B + C + D + E	4.829	0.093	0.128	0.603	0.602	0.586

Note: N = 442; A, B, C, D, and E represent WFMO, role stress, cognitive reappraisal, adaptive performance, and leader empathy, respectively. “+” indicates that two factors are in a combined state.

**Table 2:** Descriptive statistics and correlation analysis.

	M	SD	1	2	3	4	5	6	7	8	9	10
1. Years of work experience	2.850	0.948	−0.045	0.146 **	0.083	1						
2. Nature of the unit	2.110	1.133	−0.029	−0.036	0.072	−0.150 **	1					
3. WFMO	3.712	0.814	−0.049	−0.034	−0.081	−0.0260 ***	0.113 *	1				
4. Role stress	3.498	0.713	−0.100 *	0.024	−0.025	−0.327 ***	0.213 ***	0.644 ***	1			
5. Cognitive reappraisal	5.467	0.927	0.015	0.100 *	0.021	−0.210 ***	0.064	0.468 ***	0.512 ***	1		
6. Adaptive performance	3.904	0.650	−0.090	0.143 **	0.014	−0.160 ***	0.157 ***	0.437 ***	0.571 ***	0.627 ***	1	
7. Leader empathy	3.471	0.845	−0.159 **	0.069	0.021	−0.192 ***	0.159 ***	0.441 ***	0.583 ***	0.652 ***	0.656 ***	1

Note: N = 442. * indicates *p* < 0.05, ** indicates *p* < 0.01, and *** indicates *p* < 0.001, indicating significant correlation.

**Table 3:** Main effect and mediating effect test.

Variable	Adaptive Performance							Role Stress		Cognitive Reappraisal	
	Model 1	Model 2	Model 3	Model 4	Model 5	Model 6	Model 7	Model 8	Model 9	Model 10	Model 11
Gender	−0.133 *	−0.101	−0.050	−0.052	−0.133 **	−0.119 *	−0.085	−0.162 *	−0.112 *	0.000	0.050
Age	0.266 ***	0.210 ***	0.164 ***	0.168 **	0.129 **	0.134 **	0.118 **	0.123 *	0.097 *	0.229 **	0.203 **
Educational level	−0.060	−0.021	−0.029	−0.021	−0.053	−0.037	−0.035	−0.060	0.001	−0.016	0.045
Years of work experience	−0.115 ***	−0.045	0.006	0.010	−0.022	−0.004	0.027	−0.239 ***	−0.129 ***	−0.219 ***	−0.111 *
Nature of the unit	0.080 **	0.061 *	0.026	0.028	0.067 **	0.061 **	0.039	0.106 ***	0.077 **	0.029	0.000
Workplace fear of missing out		0.326 ***		0.105 **		0.137 ***	0.019		0.512 ***		0.508 ***
Role stress			0.508 ***	0.431 ***			0.286 ***				
Cognitive reappraisal					0.424 ***	0.371 ***	0.317 ***				
R^2^	0.085	0.238	0.347	0.357	0.427	0.449	0.497	0.155	0.468	0.063	0.246
ΔR^2^	0.074	0.227	0.338	0.346	0.419	0.440	0.487	0.145	0.461	0.052	0.236
F	8.071 ***	22.617 ***	38.475 ***	34.379 ***	54.067 ***	50.533 ***	53.434 ***	15.939 ***	63.905 ***	5.859 ***	23.641 ***

Note: N = 442. R^2^ represents the proportion of variance explained by the model; ΔR^2^ represents the change in R^2^ from the previous step; F represents the F-statistic for overall model fit. * *p* < 0.05, ** *p* < 0.01, *** *p* < 0.001.

**Table 4:** Mediating effect test.

	Effect	Boot SE	Boot LLCI	Boot ULCI	Effect Proportion
Role stress	0.147	0.030	0.090	0.207	45.064%
Cognitive reappraisal	0.161	0.024	0.117	0.212	49.356%
Total mediating effect	0.308	0.034	0.245	0.377	94.421%
Comparative mediation effect: Role stress-Cognitive reappraisal	0.014	0.042	−0.097	0.067	-

Note: N = 442. Bootstrap samples = 5000. Boot SE = bootstrap standard error; LLCI/ULCI = lower/upper limit of 95% confidence interval. “Effect proportion” = indirect effect/total effect × 100%. Comparative mediation effect = indirect effect (role stress) − indirect effect (cognitive reappraisal).

**Table 5:** Moderating effect of leaders’ empathy ability.

Variable	Role Stress			Cognitive Reappraisal		
	Model 12	Model 13	Model 14	Model 15	Model 16	Model 17
Gender	−0.162 *	−0.041	−0.075	0.000	0.206 **	0.187 **
Age	0.123 *	0.052	0.058	0.229 **	0.104	0.110 **
Educational level	−0.060	−0.004	−0.032	−0.016	0.032	0.013
Years of work experience	−0.239 ***	−0.105 ***	−0.059 *	−0.219 ***	−0.058	−0.032
Nature of the unit	0.106 ***	0.056 **	0.046 *	0.029	−0.045	−0.052
Workplace fear of missing out		0.394 ***	0.318 ***		0.249 ***	0.201 ***
Leader empathy		0.284 ***	0.234 ***		0.621 ***	0.614 ***
Workplace fear of missing out × Leader empathy			0.260 ***			0.140 **
R^2^	0.155	0.554	0.611	0.063	0.489	0.507
ΔR^2^	0.145	0.547	0.605	0.052	0.480	0.490
F	15.939 ***	77.110 ***	84.872 ***	5.859 ***	59.267 ***	55.570 ***

Note: ΔR^2^ = change in R-squared from the previous step; F = F-statistic for model fit. All continuous variables were mean-centered before creating interaction terms. * *p* < 0.05; ** *p* < 0.01; *** *p* < 0.001.

**Table 6:** Mediating effect of role stress and cognitive reappraisal on different leaders’ empathy ability.

Path	Leader Empathy	Effect	Boot SE	Boot LLCI	Boot ULCI
Workplace fear of missing out (X) → Role stress (M1) → Adaptive performance (Y)	eff1 (M – 1 SD)	0.021	0.013	−0.0002	0.05
	eff3 (M + 1 SD)	0.113	0.034	0.045	0.181
	eff3 − eff1	0.091	0.029	0.036	0.149
Workplace fear of missing out (X) → Cognitive reappraisal (M2) → Adaptive performance (Y)	eff1 (M – 1 SD)	0.017	0.016	−0.01	0.053
	eff3 (M + 1 SD)	0.065	0.016	0.037	0.099
	eff3 − eff1	0.048	0.018	0.014	0.084

Note: eff1 = indirect effect at low leader empathy (1 SD below mean); eff3 = indirect effect at high leader empathy (1 SD above mean); eff3 − eff1 = difference in indirect effects between high and low leader empathy; M = mean; SD = standard deviation. Bootstrap confidence intervals (LLCI = lower limit, ULCI = upper limit) based on 5000 resamples.

**Table 7:** Calibration criteria for numerical variables.

Variable Category	Variable Name	Full Membership	Crossover Point	Full Non-Membership
Result	Adaptive performance	5	3.89	2.89
Condition	Workplace fear of missing out	5	3.70	2.10
	Role stress	5	3.38	2.54
	Cognitive reappraisal	7	5.50	4.00
	Leader empathy	5	3.44	2.13

**Table 8:** Results of analysis of the necessary conditions for adaptive performance.

Variable Name	Adaptive Performance		Non-Adaptive Performance	
	Consistency	Coverage	Consistency	Coverage
Age	0.740	0.716	0.692	0.687
~Age	0.677	0.682	0.714	0.737
Nature of the unit	0.855	0.540	0.903	0.585
~Nature of the unit	0.343	0.775	0.290	0.671
Workplace fear of missing out	0.810	0.759	0.619	0.595
~Workplace fear of missing out	0.568	0.592	0.749	0.801
Role stress	0.744	0.780	0.546	0.587
~Role stress	0.606	0.565	0.796	0.761
Cognitive reappraisal	0.790	0.815	0.518	0.547
~Cognitive reappraisal	0.560	0.531	0.825	0.801
Leader empathy	0.814	0.829	0.528	0.551
~Leader empathy	0.560	0.536	0.837	0.822

Note: “~” denotes the logical relation “not”, same as below.

**Table 9:** Adaptive performance configuration pathway table.

Conditional Configuration	Adaptive Performance	
	P1	P2
Age		●
Nature of the unit		●
Workplace fear of missing out	●	⊗
Role stress	●	⊗
Cognitive reappraisal	●	●
Leader empathy	●	●
Original coverage	0.558	0.189
Unique coverage	0.395	0.027
Unique consistency rate	0.941	0.945
Overall coverage	0.585	
Overall consistency rate	0.940	

Note: ● = condition is present (core); ⊗ = condition is absent (core); blank = condition can be either present or absent. P1 = high anxiety–high stress-driven path; P2 = low anxiety–low stress compensation path.

## Data Availability

The original contributions of this study are included in the article. Further inquiries can be directed to the corresponding author.

## Abbreviations

The following abbreviations are used in this manuscript:

AI	Artificial Intelligence
WFMO	Workplace Fear of Missing Out
fsQCA	Fuzzy-Set Qualitative Comparative Analysis
COR	Conservation of Resources
SEM	Structural Equation Modeling

## Appendix A

Survey Questionnaire

Dear Participant,

Hello! Thank you for taking the time to complete this questionnaire.

The target population of this study is “new employees”—defined as those who are working for the first time and have been with their current organization for less than three years. This questionnaire aims to explore psychological and behavioral characteristics in the workplace. There are no right or wrong answers, and all data are used solely for academic research. The survey is anonymous, does not involve your personal privacy or company trade secrets, and will be kept confidential in accordance with relevant statistical laws. Please answer honestly based on your actual situation. Each question requires an answer. We sincerely appreciate your support and cooperation.

**Part 1: Demographic Information**

Please tick (✓) the box that corresponds to your information.

Gender: □ Male □ Female

Age: □ 20 years or below □ 21–25 years □ 26–30 years □ 31 years or above

Education level: □ High school or below □ Associate degree □ Bachelor’s degree □ Master’s degree or above

Tenure in current organization: □ 6 months or less □ 6–12 months □ 12–24 months □ 24–36 months

Nature of the unit: □ State-owned enterprise □ Private enterprise □ Wholly foreign-owned enterprise □ Sino-foreign joint venture □ Public institution

**Part 2: Measurement Items**

**(I) Workplace Fear of Missing Out (WFMO)**

Source: Adapted from Budnick et al. (2020).

Instructions: The following statements describe your perceptions of workplace information or opportunities. Please select the option that best fits your actual situation and tick (✓) the corresponding number. (1 = Strongly Disagree, 2 = Disagree, 3 = Neutral, 4 = Agree, 5 = Strongly Agree)

**Item**					
1. I worry that I will miss important work updates.	1	2	3	4	5
2. I worry that I will miss valuable work-related information.	1	2	3	4	5
3. I worry that I will miss important work-related messages.	1	2	3	4	5
4. I worry that I will miss important work-related information.	1	2	3	4	5
5. I worry that I will not know what is happening at work.	1	2	3	4	5
6. I worry about missing an opportunity to build an important business relationship.	1	2	3	4	5
7. I often think that I might miss an opportunity to strengthen business ties.	1	2	3	4	5
8. I often think that I might miss an opportunity to build new business relationships.	1	2	3	4	5
9. I worry that I will miss social opportunities that my coworkers have.	1	2	3	4	5
10. I worry that my coworkers might build business connections that I will not.	1	2	3	4	5

**(II) Adaptive Performance**

Source: Adapted from Griffin et al. (2007).

Instructions: Please indicate how well you adapt to work demands. (1 = Strongly Disagree, 2 = Disagree, 3 = Neutral, 4 = Agree, 5 = Strongly Agree)

**Item**					
1. I can perform the core aspects of my job well.	1	2	3	4	5
2. I can complete core tasks well using standard procedures.	1	2	3	4	5
3. I can ensure that tasks are completed correctly.	1	2	3	4	5
4. I can adapt well to changes in core tasks.	1	2	3	4	5
5. I can cope with changes in how core tasks are performed.	1	2	3	4	5
6. I can learn new skills to help adapt to changes in core tasks.	1	2	3	4	5
7. I can take the initiative to use better ways to perform core tasks.	1	2	3	4	5
8. I can think of ways to improve how core tasks are performed.	1	2	3	4	5
9. I will change the way I perform core tasks.	1	2	3	4	5

**(III) Role Stress**

Source: Adapted from Peterson et al. (1995).

Instructions: Please indicate your experience of role stress at work. (1 = Strongly Disagree, 2 = Disagree, 3 = Neutral, 4 = Agree, 5 = Strongly Agree)

**Item**					
1. There are times when I need to reduce certain aspects of my role.	1	2	3	4	5
2. I feel that my role is overloaded.	1	2	3	4	5
3. I have been given too much responsibility.	1	2	3	4	5
4. My workload is too heavy.	1	2	3	4	5
5. The amount of work I have to do affects the quality I want to maintain.	1	2	3	4	5
6. I often encounter situations where conflicting demands arise.	1	2	3	4	5
7. I receive incompatible requests from two or more people.	1	2	3	4	5
8. I have to do different things under different conditions.	1	2	3	4	5
9. I have clear, planned goals and objectives for my work.	1	2	3	4	5
10. I know exactly what is expected of me.	1	2	3	4	5
11. I know what my responsibilities are.	1	2	3	4	5
12. I am sure how much responsibility I have.	1	2	3	4	5
13. My responsibilities are not clearly defined.	1	2	3	4	5

**(IV) Leader Empathy**

Source: Adapted from X. Y. Liu and Wan (2016) for the Chinese organizational context.

Instructions: Please indicate your perception of your leader’s empathy. (1 = Strongly Disagree, 2 = Disagree, 3 = Neutral, 4 = Agree, 5 = Strongly Agree)

**Item**					
1. My leader can accurately perceive my low mood.	1	2	3	4	5
2. My leader can accurately perceive my feelings of aversion.	1	2	3	4	5
3. My leader notices my body language or nonverbal cues to understand what I am thinking.	1	2	3	4	5
4. My leader can accurately perceive my fear.	1	2	3	4	5
5. My leader can easily tell whether I am listening seriously.	1	2	3	4	5
6. Before making decisions, my leader tries to see everyone’s position.	1	2	3	4	5
7. My leader sees things from my perspective to better understand the situation.	1	2	3	4	5
8. My ideas are easily recognized by my leader.	1	2	3	4	5
9. Before criticizing others, my leader imagines how they would feel in that situation.	1	2	3	4	5
10. My leader understands the purpose of my actions.	1	2	3	4	5
11. My leader can detect deficiencies in work.	1	2	3	4	5
12. My leader offers help when I am helpless at work.	1	2	3	4	5
13. When seeing me treated unfairly, my leader shows sympathy.	1	2	3	4	5
14. My leader allows me to solve work problems in my own way.	1	2	3	4	5
15. When I look unwell, my leader cares about why.	1	2	3	4	5
16. My leader does their best to make it easier for me to carry out my work.	1	2	3	4	5

**(V) Cognitive Reappraisal**

Source: Wang et al. (2007); revised from Gross and John (2003).

Instructions: Please indicate how you regulate your emotions at work. (1 = Strongly Disagree, 2 = Very Much Disagree, 3 = Slightly Disagree, 4 = Neutral, 5 = Slightly Agree, 6 = Very Much Agree, 7 = Strongly Agree)

**Item**							
1. When I want to feel more positive emotions (e.g., happiness or pleasure), I change the way I think about the situation.	1	2	3	4	5	6	7
2. When I want to feel less negative emotions (e.g., sadness or anger), I change the way I think about the situation.	1	2	3	4	5	6	7
3. When facing stressful emotions, I try to think about them in a way that helps me stay calm.	1	2	3	4	5	6	7
4. When I want to feel more positive emotions, I change my perspective on the situation.	1	2	3	4	5	6	7
5. I control my emotions by changing the way I think about the situation.	1	2	3	4	5	6	7
6. When I want to feel less negative emotions, I change the way I think about the situation.	1	2	3	4	5	6	7
